# Effect of *Pranayama* (voluntary regulated breathing) and *Yogasana* (yoga postures) on lipid profile in normal healthy junior footballers

**DOI:** 10.4103/0973-6131.72633

**Published:** 2010

**Authors:** BK Acharya, AK Upadhyay, Ruchita T Upadhyay, A Kumar

**Affiliations:** Department of Research and Development, Divya Yog Mandir Trust (SIRO), Patanjali Yog Peeth, Haridwar, India; 1 Yog Education and Training, Divya Yog Mandir Trust (SIRO), Patanjali Yog Peeth; Haridwar, India; 2 Department of Research and Development, Patanjali Ayurved Limited, D-38 Industrial Area, Haridwar, India E-mail: avnishdr@yahoo.co.in

There are many styles of *Pranayama* (Voluntary Regulated Breathing) and *Yogasana* (Yoga Postures) that range from very dynamic, active movements that go from one posture to another (and result in a thorough aerobic workout) to more slow-paced practices that hold postures for several minutes and form an intense strength training and balanced workout. Twenty male junior footballers younger than 15 years of age, belonging to the Mohun Bagan Athletic Club, Kolkata, were selected for the study at Haridwar. They had to play in a Football Cup organized in UK and they were here to practice yoga sequences taught by Swami Ramdevji.[1–3] They were of age 14.65±0.58 years and none of them had a history of lipid metabolism disorders. All the footballers were healthy with no history of smoking or alcohol consumption. The scope and objectives of the present study were explained to the subjects and their written consent was obtained for participation in the study. The institutional ethical committee had approved the study protocol and design. The subjects were asked to follow their routine diet and exercise pattern during the period of study. None of the subjects were exposed to *yogic* practices before this *yoga* training session. There was a significant reduction in the levels of serum cholesterol, Low-density lipoprotein (LDL) cholesterol, serum triglycerides, and very-low-density lipoprotein (VLDL)-cholesterol at the end of the *yoga* session. The results indicated that the fasting blood sugar (FBS) level was positively elevated in junior footballers. This demonstrated that *Pranayama* and *Yogasana* were helpful in regulating sugar level also [Table 1 and Figure 1].

**Figure 1:**
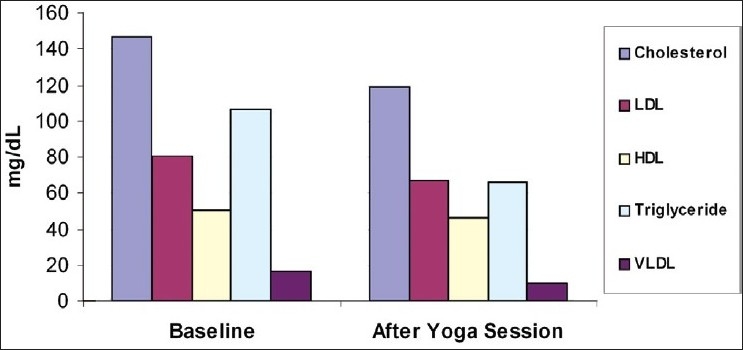
Change in lipid profile after yoga session

**Table 1:** Lipid profile in subjects before and after yoga practices

Parameters	Baseline	After yoga session
FBS	80.94±14.38	94.39±7.02***
Cholesterol	147.00±26.50	118.65±18.22***
LDL	80.70±13.67	66.50±12.59**
HDL	50.70±12.55	46.15±07.60
Triglyceride	106.40±30.46	66.10±17.60***
VLDL	16.27±04.64	10.12±02.71***
TC/HDL ratio	02.75±00.33	02.55±00.27*

Values expressed as mean±SD, significant at ^***^P<0.001,

** *P*<0.01,

*P*<0.05

The present study demonstrates the efficacy of SRY (Swami Ramdev *Yoga*)- *Pranayama* and *Yogasana* sequences on blood lipid profiles in normal healthy footballers. *Pranayama* and *Yogasana* can be used as supportive therapy in patients with lipid disorders, heart diseases, hypoglycemia, and so on. There is a need for conducting the experiments on a larger number of participants, to explore the results and mode of action.

## References

[1] Acharya Balkrishna (2007-6). Yog-In Synergy with Medical Science.

[2] Swami Ramdev (2005-3). Yog-Its Philosophy and Practice.

[3] Swami Ramdev (2005-3). Pranayama Rahasya.

